# Long-Term Diabetic Microenvironment Augments the Decay Rate of Capsaicin-Induced Currents in Mouse Dorsal Root Ganglion Neurons

**DOI:** 10.3390/molecules24040775

**Published:** 2019-02-21

**Authors:** Xingjuan Chen, Yaqian Duan, Ashley M. Riley, Megan A. Welch, Fletcher A. White, Maria B. Grant, Alexander G. Obukhov

**Affiliations:** 1 Department of Cellular and Integrative Physiology, Indiana University School of Medicine, Indianapolis, IN 46202, USA; xingjuanchen@yahoo.com (X.C.); yaqduan@umail.iu.edu (Y.D.); ashlrile@indiana.edu (A.M.R.); megwelch@imail.iu.edu (M.A.W.); 2 Department of Ophthalmology and Visual Sciences, School of Medicine, University of Alabama at Birmingham, Birmingham, AL 35294, USA; mariagrant@uabmc.edu; 3 Department of Anesthesia, Indiana University School of Medicine, Indianapolis, IN 46202, USA; fawhite@iu.edu; 4 Stark Neurosciences Research Institute, Indiana University School of Medicine, Indianapolis, IN 46202, USA; 5 Indianapolis VA Medical Center, Indianapolis, IN, 46202, USA

**Keywords:** calcium influx, DRG neurons, *Ins2^+/Akita^* mouse, ROS, capsaicin, TRPV1

## Abstract

Individuals with end-stage diabetic peripheral neuropathy present with decreased pain sensation. Transient receptor potential vanilloid type 1 (TRPV1) is implicated in pain signaling and resides on sensory dorsal root ganglion (DRG) neurons. We investigated the expression and functional activity of TRPV1 in DRG neurons of the *Ins2^+/Akita^* mouse at 9 months of diabetes using immunohistochemistry, live single cell calcium imaging, and whole-cell patch-clamp electrophysiology. 2′,7′-Dichlorodihydrofluorescein diacetate (DCFH-DA) fluorescence assay was used to determine the level of Reactive Oxygen Species (ROS) in DRGs. Although TRPV1 expressing neuron percentage was increased in *Ins2^+/Akita^* DRGs at 9 months of diabetes compared to control, capsaicin-induced Ca^2+^ influx was smaller in isolated *Ins2^+/Akita^* DRG neurons, indicating impaired TRPV1 function. Consistently, capsaicin-induced Ca^2+^ influx was decreased in control DRG neurons cultured in the presence of 25 mM glucose for seven days versus those cultured with 5.5 mM glucose. The high glucose environment increased cytoplasmic ROS accumulation in cultured DRG neurons. Patch-clamp recordings revealed that capsaicin-activated currents decayed faster in isolated *Ins2^+/Akita^* DRG neurons as compared to those in control neurons. We propose that in poorly controlled diabetes, the accelerated rate of capsaicin-sensitive TRPV1 current decay in DRG neurons decreases overall TRPV1 activity and contributes to peripheral neuropathy.

## 1. Introduction

Individuals diagnosed with diabetes mellitus are at increased risk of developing microcomplications of the disease, including diabetic retinopathy, renal failure, and peripheral neuropathy. Peripheral neuropathy is one of the most common complications of end-stage diabetes, which manifests as symmetrically decreased pain sensation in the lower extremities and is associated with a high incidence of foot ulceration. A retrospective study in 2015 estimated the prevalence of peripheral neuropathy to be about 30% in diabetic patients [1], while other studies have reported up to 80% [2,3]. 

Despite the high prevalence of this condition, the mechanisms underlying this dysfunction are poorly understood, limiting the development of new therapeutic strategies. The peripheral processes of dorsal root ganglion (DRG) neurons innervate the cutaneous surface of the foot. The transient receptor potential vanilloid type 1 (TRPV1) channel is a Ca^2+^ permeable plasma membrane cation channel that can be activated by heat, acid, and capsaicin [4,5,6] and is expressed robustly in the nociceptive sensory neurons of DRG [7,8]. TRPV1 is known to be upregulated in a number of clinical disease-associated pain conditions [9,10,11,12]. Animal models of induced diabetes have demonstrated increased TRPV1 expression that correlates to hyperalgesia and decreased TRPV1 expression in hypoalgesia [13], reflecting earlier and later manifestations of diabetic neuropathy, respectively. 

Elevated Reactive Oxygen Species (ROS) are associated with the pathogenesis of diabetes and diabetic complications [14]. Hyperglycemia is the major trigger of ROS accumulation in diabetes. Notably, TRPV1 channels are sensitive to ROS. It has been demonstrated that H_2_O_2_ activates TRPV1 and potentiates the capsaicin-induced TRPV1 currents after short-term treatment and that the prolonged treatment with H_2_O_2_ reduced the capsaicin-induced TRPV1 current amplitude in human embryonic kidney (HEK) cells [15,16]. ROS are known to be elevated in hereditary model of diabetes, such as *Ins2^+/Akita^* mice, exhibiting hyperglycemia and hypoinsulinemia, but no obesity [17].

In this study, we investigated the function activity of TRPV1 in DRG neurons from long-term diabetic *Ins2^+/Akita^* mice to determine whether TRPV1 activity is modulated under a diabetic environment. We also assessed short-term changes in high glucose-induced ROS accumulation in DRG neurons. 

## 2. Results

### 2.1. Changes in Blood Glucose Level, Body Weight

*Ins2^+/Akita^* mouse is a hereditary model of diabetes [18,19,20]. In this study, only male wild-type and *Ins2^+/Akita^* mice were used as the male *Ins2^+/Akita^* mice show more profound diabetic phenotype than the female mice [19,20]. Mice were observed for a period of 36 weeks after the onset of diabetes. Blood glucose levels were significantly elevated within 6 weeks and remained elevated (520 ± 21.4 mg/dL, *n* = 4) as compared to control non-diabetic mice (216 ± 27.2 mg/dL, *n* = 4). As the disease progressed (9 months after the onset of diabetes), the overall body weight of the *Ins2^+/Akita^* mice (23.1 ± 1.8 g, *n* = 6) was much lower when compared to their wild-type littermates (40.7 ± 1.7 g, *n* = 6).

### 2.2. Increased Positive TRPV1 Staining DRGs Neurons in Ins2^+/Akita^ Mice

**Figure 1:**
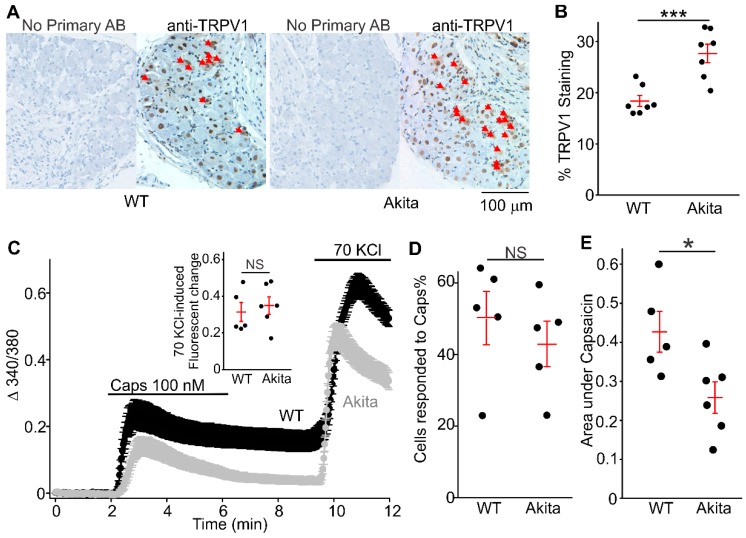
Transient receptor potential vanilloid type 1 (TRPV1) channel expression and capsaicin-evoked Ca^2+^ transients in dorsal root ganglion (DRG) neurons from wild-type and *Ins2^+/Akita^* mice at 9 months of diabetes. (**A**) Sample images of immunohistochemistry showing the expression of TRPV1 in DRG neurons of wild-type and *Ins2^+/Akita^* mice (Akita). Arrows indicate neurons with positive TRPV1 staining. (**B**) Percentage of TRPV1-positive neurons in wild-type and *Ins2^+/Akita^* (Akita) mice. The percentage of TRPV1-positive neurons was determined in six segments of each mouse DRG section and averaged. The dots represent the percentage determined for each wild-type or *Ins2^+/Akita^* mouse (*n* = seven mice for each condition; *p* = 0.0008). (**C**) Sample traces of time-course of capsaicin (Caps)-and 70 KCl-induced [Ca^2+^]_i_ rises in DRG neurons isolated from one control or one Akita mouse. The experiments were repeated five to six times in DRG neurons isolated from additional five to six mice. Solid lines are averaged [Ca^2+^]_i_ changes. The vertical lines represent SEM. The compounds were applied at the times indicated with the horizontal bars. The right inset shows the summary of fluorescence changes induced by 70 KCl. Each dot represents the averaged fluorescence change value determined during a fluorescence imaging experiment on a coverslip with 20 to 150 DRG neurons (*n* = 321–335 cells from five to six mice for each condition; *p* = 0.62). (**D**) The summary data of the average percentage of DRG neurons sensitive to capsaicin. Each dot represents the averaged percentage value determined in the tested cells from each mouse (*n* = five mice; *p* = 0.47). (**E**) Summary data of the areas under the response curves for **C**. Each dot represents the averaged area values determined for the tested cells on each coverslip. There were 20 to 150 isolated DRG neurons per coverslip from one mouse assessed in each experiment. Five wild-type and six *Ins2^+/Akita^* mice were used for these experiments. Only the neurons exhibiting Ca^2+^ increases in the presence of 70 KCl were included in the statistics. The *t* test was used to determine whether there was a significant difference between the tested groups (*p* = 0.027). * indicates *p* < 0.05 and *** indicates *p* < 0.001.

Studies using *Ins2^+/Akita^* mice reportedly exhibit impaired thermal nociception at an age of 12 weeks [21]. Channels thought to contribute to these changes include TRPV1, which is known to be upregulated in several clinical disease-associated pain conditions [9,10,11,12]. Consistently, Figure 1A,B shows that a greater number of neurons are TRPV1 positive in *Ins2^+/Akita^* DRGs (27.3 ± 1.83%, *n* = 7, 42 section slices from seven *Ins2^+/Akita^* mice) when compared with wild-type DRGs (18.0 ± 1.56%, *n* = 7, 42 section slices from seven wild-type mice). 

### 2.3. Capsaicin-Evoked [Ca^2+^]_i_ Responses in Wild-Type and Ins2^+/Akita^ DRGs

Capsaicin, a member of the vanilloid family, binds to TRPV1 and depolarizes isolated sensory neurons. In Figure 1C,D, we can see that the percentage of isolated DRG neurons (cultured overnight after isolation) sensitive to capsaicin (100 nM) in the *Ins2^+/Akita^* group (42.7 ± 6.5%, 321 cells from six mice) was not different from the wild-type group (49.8 ± 7.2%, 337 cells from five mice). However, the capsaicin-induced intracellular calcium increases in *Ins2^+/Akita^* neurons (ΔF340/F380*s = 0.26 ± 0.04, *n* = 6, 321 cells from six mice) were significantly smaller than those in wild-type neurons (ΔF340/F380*s = 0.43 ± 0.05, *n* = 5, 337 cells from five mice). The intracellular calcium increases induced by 70 KCl was not different between the *Ins2^+/Akita^* and wild-type (right inset in Figure 1C). These data suggest that TRPV1 function may be impaired in diabetic *Ins2^+/Akita^* DRG neurons. 

### 2.4. Voltage-Gated Calcium and TRPV1 Currents in Wild-Type and Ins2^+/Akita^ DRG Neurons

**Figure 2:**
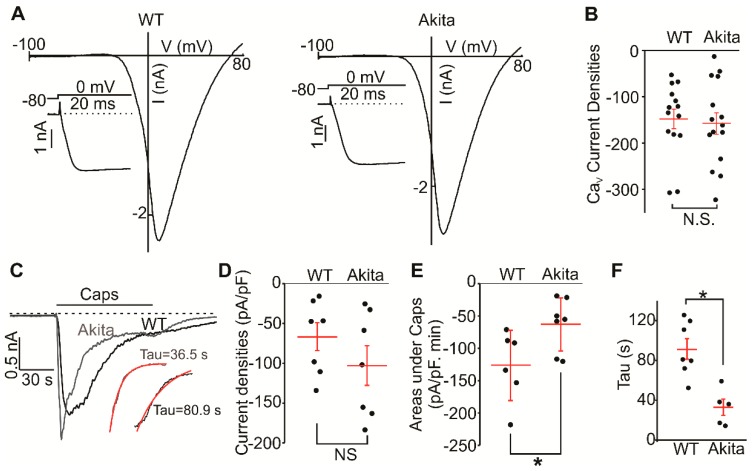
Voltage-gated calcium and TRPV1 currents in DRG neurons from wild-type and *Ins2^+/Akita^* mice. (**A**) Sample traces of current-voltage relationships acquired during the voltage ramps from −100 mV to +80 mV in wild-type (left) and *Ins2^+/Akita^* (Akita) DRG (right) neurons. The insets show the voltage-gated calcium currents activated by depolarizing pulses to 0 mV. (**B**) The summary data of the current densities recorded in wild-type and *Ins2^+/Akita^* (Akita) DRG neurons (*p* = 0.75). (**C**) Sample current traces obtained during the applications of 2 µM capsaicin (Caps) in wild-type and *Ins2^+/Akita^* (Akita) DRG neurons at a holding potential of −60 mV. The insets showed the fitted curves. A standard exponential function was used to fit to the current decay (red curve). (**D**) The summary data of capsaicin (Caps)-induced current densities for C (*p* = 0.27). (**E**) A comparison of the areas under the curves in the presence of capsaicin (Caps) in wild-type and *Ins2^+/Akita^* (Akita) DRG neurons (*p* = 0.036). (**F**) The summary data of the current decay tau values from wild-type and *Ins2^+/Akita^* DRGs. The *t* test was used to determine whether there was a significant difference between the tested groups (*p* = 0.002). * indicates *p* < 0.05.

Ion channels, such as voltage-gated potassium channels, are known to be altered in neurons derived from diabetic rodents [22]. To determine the degree to which voltage-gated Ca^2+^ channels were affected by a diabetic environment, we measured voltage-gated Ca^2+^ currents in small-and mid-sized *Ins2^+/Akita^* and wild-type DRG neurons. The cells were cultured overnight before the whole cell patch-clamp experiments. We found that the voltage-gated Ca^2+^ current amplitudes were not different in *Ins2^+/Akita^* (−157.9 ± 23.4 pA/pF, *n* = 15) and wild-type (−147.7 ± 21.1 pA/pF, *n* = 15, Figure 2A,B) groups. This was in accordance with our Ca^2+^-imaging experiments where 70 KCl induced similar calcium changes in *Ins2^+/Akita^* and wild-type neurons (Figure 1C, inset). We next assessed the function of TRPV1 using capsaicin (2 μM) applications. Figure 2C,D illustrate that the average peak TRPV1 current amplitudes were not significantly different in *Ins2^+/Akita^* neurons (−103.0 ± 24.8 pA/pF, *n* = 7) and wild-type neurons (−67.8 ± 17.5 pA/pF, *n* = 7). However, the TRPV1 currents decayed faster after reaching the peak value in *Ins2^+/Akita^* neurons than wild-type (Figure 2C insets). To evaluate the current decay, the areas under the curve of capsaicin-induced currents were calculated. Figure 2E shows that the *Ins2^+/Akita^* neurons had significantly smaller areas under the curve during capsaicin application (63.0 ± 15.5, *n* = 7) than wild-type neurons (126.1 ± 22.3, *n* = 7). Although *Ins2^+/Akita^* and wild-type neurons had similar capsaicin-induced peak currents, the currents decayed much faster (Figure 2F, time constant: 91.5 ± 10.3 s vs 32.9 ± 8.1 s, respectively).

### 2.5. High Glucose Decreased the Capsaicin-Evoked [Ca^2+^]_i_-Responses and Increased Intracellular ROS Accumulation

**Figure 3:**
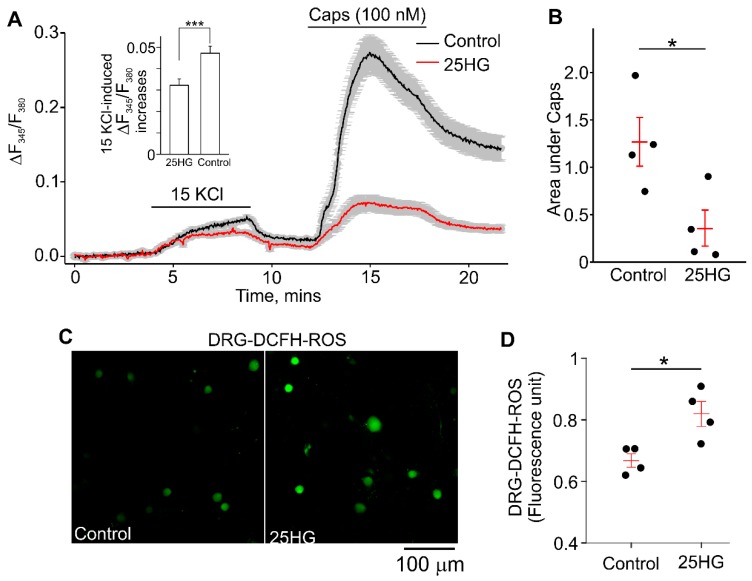
Effect of high glucose treatment on capsaicin (Caps)-induced [Ca^2+^]_i_ rises and intracellular reactive oxygen species (ROS) accumulation in primary cultured DRG neurons. (**A**) Sample traces of time-course of [Ca^2+^]_i_ changes in cultured DRG neurons isolated from one mouse (*n* = 91 neurons for control and *n* = 82 neurons for 25 HG). DRG neurons were treated with 5.5 mM (control, black) and 25 mM glucose (25HG, red) for 7 days. Solid and broken lines are averaged [Ca^2+^]_i_ changes. The vertical lines represent SEM. The compounds were applied at the times indicated with the horizontal bars. The inset shows a comparison of 15 KCl-induced [Ca^2+^]_i_ changes at the peak of the response in the control and 25HG groups. The data were averaged over five data points (*n* = 91 neurons for control and *n* = 82 neurons for 25 HG; Mann–Whitney Rank Sum Test, *p* ≤ 0.001). (**B**) Summary data of the areas under the capsaicin-induced [Ca^2+^]_i_ response curves (sample curves are shown in A). The dots represent the averaged area under the curves determined for each mouse. There were 20 to 150 isolated DRG neurons per coverslip from one mouse assessed in each experiment. The experiments were repeated four times (*n* = four mice). The *t* test was used to determine whether there was a significant difference between the tested groups (*p* = 0.029). (**C**) The representative images of ROS detection by DCFH in DRG neurons treated with 5.5 mM (control) or 25 mM glucose (25HG) for 7 days. (**D**) Summary of data shown in **C**. ROS production was assessed in high glucose-treated DRG neurons (25HG) and control DRG neurons by measuring the DCFH fluorescence intensity. The dots represent the averaged values of DCFH fluorescence intensities calculated for each mouse. There were 77 to 256 isolated DRG neurons per coverslip from one mouse assessed in each experiment. The experiments were repeated four times (*n* = four mice). The *t* test was used to determine whether there was a significant difference between the tested groups (*p* = 0.017). * indicates *p* < 0.05; *** indicates *p* < 0.001.

We next investigated the effect of treatment with 25 mM glucose on capsaicin-induced [Ca^2+^]_i_ increases and ROS production in wild-type DRG neurons to ascertain whether there is a correlation between the elevated glucose, ROS, and TRPV1 function. Isolated DRG neurons from wild-type C57BL/6J mice were treated with 25 mM glucose (25HG) for 7 days to mimic the diabetic environment. The percentage of cultured DRG neurons sensitive to capsaicin (100 nM) in the 25HG treated group was not different from that observed in the control group. The DRG neurons treated with 25HG had significantly smaller intracellular calcium increases evoked by 100 nM capsaicin than control neurons treated with only 5.5 mM glucose (Figure 3A,B, ΔF340/F380*s = 0.36 ± 0.19 vs ΔF340/F380*s = 1.27 ± 0.26, respectively). Notably, the 15 mM KCl-induced [Ca^2+^]_i_ responses were smaller in DRG neurons treated with 25 mM glucose compared to control DRG neurons (Figure 3A, inset). The cytoplasmic redox state was monitored using a ROS sensitive dye, 2′,7′-dichlorodihydrofluorescein diacetate (DCFH-DA) [23,24], in cultured neurons under control and 25HG conditions. The DCFH-DA fluorescence intensity in DRG neurons from 25HG-treated group was significantly increased as compared to that in the control group (Figure 3C,D, 25HG: 0.83 ± 0.01, *n* = 585; control: 0.66 ± 0.01, *n* = 765). These data suggest that the functional change of TRPV1 in short-term diabetic conditions correlates with the increased levels of cytoplasmic ROS.

## 3. Discussion

The major novel finding in this study is the fact that capsaicin-induced currents exhibit a markedly accelerated rate of decay in *Ins2^+/Akita^* DRG neurons at 9 months of diabetes compared to DRG neurons isolated from control DRGs. In addition, our data show that the TRPV1 expression is increased in end-stage diabetes. However, the capsaicin-induced calcium responses are decreased, suggesting the impaired TRPV1 function in diabetic DRG neurons. Consistent with previous reports [25,26], we observed an increased production of ROS in DRG neurons cultured in the presence of 25 mM of glucose, simulating a hyperglycemic state. The electrophysiological recordings do not show impairment in the function of voltage-gated Ca^2+^ channels in *Ins2^+/Akita^* DRG neurons compared to control DRG neurons. 

Several studies, using streptozotocin (STZ)-treated mice, a model of type 1 diabetes, reported that in DRG neurons, elevated TRPV1 expression is associated with hyperalgesia and decreased TRPV1 expression is linked to loss of pain sensation (hypoalgesia) [13,27]. There are several mechanisms underlying the hyperalgesic phenotype in short-term diabetes, involving long non-coding RNA BC168687 [28] and protein kinase C (PKC) activation [29]. However, it remains unclear how TRPV1 function and expression are modulated in hypoalgesia associated with end-stage diabetes.

Our data suggest that the percentage of TRPV1-positive neurons is increased in intact DRGs from 9-month diabetic *Ins2^+/Akita^* mice as determined by immunohistochemistry, whereas in cultured DRG neurons, isolated from the same animals, the percentage of capsaicin-sensitive neurons is not different between the diabetic and control groups as ascertained using the calcium imaging assay (Figure 1). Moreover, we demonstrate that the percentage of capsaicin-sensitive neurons is higher in the primary cultures than expected from the immunohistochemistry data. This apparent discrepancy may result from several factors. Firstly, survival of certain populations of neurons may be variably affected during the DRG dissociation procedure and short-term culture. Secondly, the TRPV1 protein is known to be expressed on both the plasma membrane and the endoplasmic reticulum in sensory neurons [30], whereas the calcium imaging assay evaluates TRPV1 function only in the plasma membrane at nanomolar concentrations of capsaicin. The degree to which the observed protein changes affect mechanical and thermal nociception is unknown. However, it was reported that 5-month diabetic *Ins2^+/Akita^* mice already presented with impaired mechanical and thermal nociception [21]. This is consistent with our calcium imaging and electrophysiology data revealing that TRPV1 activity decays much faster in *Ins2^+/Akita^* DRG neurons at 9 months of diabetes. 

A recent study reported a RAGE (receptor for Advanced Glycation End-products)-dependent potentiation of TRPV1 currents in sensory neurons exposed to high glucose, implicating RAGE in modulating TRPV1 [25], with high glucose treated DRG neurons showing potentiated capsaicin-evoked TRPV1 currents and elevated intracellular ROS accumulation [25]. In our study, high glucose treatment of DRG neurons also resulted in an increase in ROS production (Figure 3C). However, we observed reduced capsaicin-stimulated responses in high glucose treated DRG neurons. We also did not observe the potentiated peak amplitudes of capsaicin-induced currents in *Ins2^+/Akita^* DRG neurons at 9 months of diabetes compared to control DRG neurons (Figure 2C–E), and we found that the currents exhibited much faster decays (Figure 2F). Consistently, smaller capsaicin-induced intracellular calcium changes were observed in *Ins2^+/Akita^* DRG neurons compared to wild-type neurons (Figure 1C,E). In the previously cited study, data was obtained from neonatal or 2 to 3-month-old mice, while we used >9 month-old mice. Therefore, we cannot exclude that our differing observations may be due to animal age. Moreover, ligands that can accompany diabetic conditions and activate RAGE, such as the inflammatory cytokine high-mobility group box-protein 1 (HMGB1, also known as amphoterin) and methylglyoxal, may contribute to pain behavior in diabetic neuropathy via TRPV1 or transient receptor potential ankyrin 1 (TRPA1)-dependent mechanisms [31,32].

The observed facilitated decay of TRPV1 activity reported in this study may be due to accelerated desensitization of the channel. The mechanisms of the TRPV1 desensitization are diverse and may involve multiple intracellular signaling pathways, and the process is often Ca^2+^-dependent [33]. Notably, long capsaicin exposure induced TRPV1 endocytosis and degradation and decrease in TRPV1 activity [34]. It is well known that phosphorylation of TRPV1 sensitizes the channel [35]. Consistently, dephosphorylation of p-TRPV1 by the phosphatase calcineurin was shown to lead to TRPV1 desensitization [36]. Several other mechanisms of TRPV1 desensitization were also proposed such as binding of calmodulin [37] and the depletion of phosphatidylinositol 4,5-diphosphate from the plasma membrane [38]. Future experiments will be needed to establish whether these processes modulate TRPV1 activity in the setting of end-stage diabetes.

Chuang and Lin reported that H_2_O_2_ activates TRPV1 and potentiates the capsaicin-induced TRPV1 currents [15], whereas DelloStritto et al. (2016, [16]) demonstrated that the prolonged treatment with H_2_O_2_ reduced the capsaicin-induced TRPV1 current amplitude in HEK cells. Thus, ROS might modulate TRPV1 activity. The mechanism for H_2_O_2_-dependent modulation of TRPV1 function remains to be determined. Ca^2+^ influx through TRPV1 may facilitate Ca^2+^-dependent desensitization of the channel [39], signifying that H_2_O_2_ may be a possible factor in the inhibitory effect of diabetic environment on TRPV1 through a Ca^2+^ dependent mechanism. Alternatively, Chuang and Lin (2009) reported [15] that marked H_2_O_2_-dependent sensitization of TRPV1 might be due to the formation of disulfide bonds, likely between the N-terminal C158 and the C-terminal C783. Further experiments will be needed to determine whether Ca^2+^ influx or H_2_O_2_–dependent specific cysteine residue oxidation and reversible disulfide bond formation [40] underlies the accelerated TRPV1 current decay observed in Akita DRG neurons at 9 months of diabetes. Long-term in vivo treatment with antioxidants in diabetic Akita mice will be needed to provide evidence whether an association exists between long-term ROS accumulation and the accelerated kinetics of TRPV1 current decay observed in end-stage diabetes. 

A subset of TRPV1-positive DRG neurons co-expresses the TRPA1 channel. It has been reported that TRPA1 and TRPV1 channels can interact physically and functionally [41,42,43]. Notably, heterologous desensitization between these two channels may occur in DRG neurons [41]. Since H_2_O_2_ also activates TRPA1 [44], it is possible that ROS-dependent activation of TRPA1 channels followed by heterologous TRPV1 desensitization may lead to accelerating the TRPV1 current decay rate. On the other hand, a recent study [43] provided evidence that in TRPA1-positive DRG neurons, the decay rate of capsaicin-induced currents was apparently slower due to cross-activation of TRPA1 by capsaicin-induced calcium influx, suggesting that TRPA1 protein depletion may result in accelerating the decay rate of capsaicin-induced currents. Thus, it remains to be determined whether cross-desensitization or cross-activation between TRPA1 and TRPV1 would prevail in regulating TRPV1 current kinetics in diabetes.

In addition to being activated by low pH and capsaicin, TRPV1 is also activated by heat. A recent study [21] found reduced behavioral sensitivity to noxious heat associated with a reduced temperature-activated [Ca^2+^]_i_-response amplitude in DRG neurons from *Ins2^+/Akita^* mice at 20 weeks of diabetes. However, the authors reported that no difference was observed in capsaicin-evoked [Ca^2+^]_i_ responses in wild-type and *Ins2^+/Akita^* DRG neurons. We found decreased overall capsaicin-induced [Ca^2+^]_i_ increases in diabetic *Ins2^+/Akita^* DRG neurons compared to wild-type DRG neurons. This may be because our *Ins2^+/Akita^* diabetic mice had 9 months of diabetes, which is more comparable to end-stage diabetes. 

**Figure 4:**
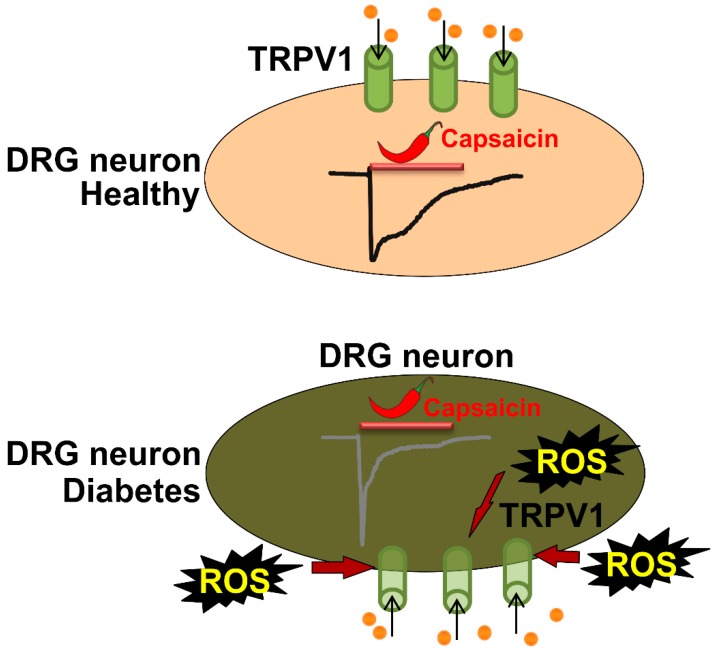
Diagram shows the putative changes in TRPV1 functional activity in DRG neurons. The upper panel shows a healthy DRG neuron and the lower panel shows a DRG neuron in late-stage diabetes. Sample capsaicin-activated TRPV1 current traces are demonstrated for each condition. High glucose induces increased ROS production in DRG neurons. Accelerated current decay is a characteristic feature of capsaicin-activated TRPV1 currents in DRG neurons isolated from Akita mice at 9 months of diabetes.

In summary, we found that long-term diabetic *Ins2^+/Akita^* DRG neurons exhibited decreased capsaicin-induced Ca^2+^ transients that decayed faster. High glucose-treated DRG neurons produced increased amounts of ROS. Although ROS is known to modulate TRPV1 function [15,45], it remains unclear whether ROS accumulation in Akita mice directly or indirectly results in TRPV1 function impairment. We propose that the accelerated decay rate of capsaicin-induced TRPV1 activity (Figure 4) may contribute to reduced pain sensation in end-stage diabetes.

## 4. Materials and Methods

### 4.1. Animals

All animal procedures were performed in accordance to the National Institutes of Health (NIH) guide and were approved by the Indiana University School of Medicine Institutional Animal Care and Use Committee (Animal Protocol #11165). Mice were euthanized using isoflurane anesthesia followed by decapitation. C57BL/6J wild-type and *Ins2^+/Akita^* mice were obtained from The Jackson Laboratory (Bar Harbor, ME, USA) and bred in the animal facilities at the Indiana University School of Medicine. 

### 4.2. Dorsal Root Ganglia (DRG) Neuron Isolation

DRG neurons were isolated from control C57BL/6J wild-type or *Ins2^+/Akita^* mice using methods described previously [46]. Briefly, thoracic and lumbar (T1-L5) DRGs were collected and placed into cold Phosphate Buffered Saline (PBS) containing no Ca^2+^ or Mg^2+^. DRGs were digested in collagenase P (1 mg/mL, from Clostridium histolyticum, Roche, Indianapolis, IN, USA) solution in Hank’s Balanced Salt medium supplemented with 0.2 mM CaCl_2_, 0.12% bovine serum albumin (BSA), and the Trypsin Inhibitor from soybean (0.1 mg/mL, Roche, Indianapolis, IN, USA) for 30 to 60 min. DRG neurons were dispersed by trituration and then plated onto Growth Factor Reduced Matrigel (Life Technologies, Grand Island, NY, USA)-coated 15 mm glass coverslips and cultured for 24 h in Minimum Essential Medium (Life Technologies) supplemented with 0.2% BSA, 100 U/mL penicillin, 100 μg/mL streptomycin, and 20 ng/mL NGF-2.5S (BD Biosciences, Bedford, MA, USA) at 37 °C in a water-jacketed 5% CO_2_ incubator. 

### 4.3. Whole-Cell Patch-Clamp Recordings

The capsaicin-activated TRPV1 currents were recorded from DRGs using an Axopatch 200B amplifier (Molecular Devices, San Jose, CA, USA) as indicated elsewhere [46,47,48,49,50]. Briefly, the analog data were digitized using an analog-digital converter Digidata 1440a at a sampling rate of 1 kHz. The currents were filtered at 3 kHz. Series resistance compensation was set to 50%. The external solution contained (in mM): 145 NaCl, 2.5 KCl, 1.2 CaCl_2_, 1 MgCl_2_, 10 4-(2-hydroxyethyl)-1-piperazineethanesulfonic acid (HEPES), and 5.5 glucose (pH 7.2). The intracellular solution contained the following (in mM): 125 CsMeSO_3_, 3.77 CaCl_2_, 2 MgCl_2_, 10 EGTA (100 nM free Ca^2+^), and 10 HEPES (pH 7.2). For TRPV1 current or voltage-gated calcium current recording in DRG neurons, 3 mM ATP-Mg was added into the intracellular solution. An episodic stimulation voltage protocol was used to record TRPV1 currents. Cells were voltage-clamped at a potential of −60 mV and voltage ramps from −100 to +100 mV were applied at 2-s intervals. The voltage protocol for voltage-gated calcium current was as follows: Cells were voltage-clamped at a holding potential of −80 mV, a 20 ms depolarizing pulse to 0 mV was used to transiently activate the voltage-gated calcium channels, and then a voltage ramps from −100 to +80 mV was applied. The voltage protocol was repeated with an interval of 15 s throughout the experiment. During the voltage-gated calcium current recordings, Ba^2+^ was used as a surrogate for Ca^2+^. The Ba^2+^ extracellular solution contained (in mM): 135 *N*-methyl-d-glucamine (NMDG)-Cl, 10 BaCl_2_, 10 HEPES, and 5.5 glucose (pH 7.2). The pCLAMP 10 software package (Molecular Devices, San Jose, CA, USA) was used for acquisition control and data analyses. The current density was calculated by dividing the peak current by the cell capacitance. The data was obtained from 7 to 15 DRG neurons from 4 to 6 mice in each group. Data were fitted to a single exponential function. All of the experiments were performed at room temperature (22 to 25 °C).

### 4.4. Intracellular [Ca^2+^]_i_ Measurements

Fluorescence imaging experiments were performed as described previously [46,51]. Briefly, DRG neurons were loaded with Fura-2AM (4 µM) in Ca/Mg-PBS for one hour followed by an additional 30-minute incubation in Ca/Mg-PBS without Fura-2AM. A Till Photonics single-cell fluorescence imaging system was used to monitor intracellular Ca^2+^ changes in single Fura-2-loaded DRG neuron. The standard extracellular solution contained (in mM): 145 NaCl, 2.5 KCl, 1.2 CaCl_2_, 1 MgCl_2_, 10 HEPES, and 5.5 glucose (pH 7.2). 70 mM KCl (70 KCl) solution contained (in mM): 77.5 NaCl, 70 KCl, 1.2 CaCl_2_, 1 MgCl_2_, 10 HEPES, and 5.5 glucose (pH 7.2). The 15 mM KCl (15KCl) was obtained by mixing the standard extracellular solution and 70 KCl solution at a ratio of 11/2.5. The cells were superfused continuously with the test solutions at a rate of 1.5 mL/min. DRG neurons were identified by their appearance (round cells with processes). Only DRG neurons responding with pronounced Ca^2+^ influx upon KCl-induced depolarization were used for analyses. There were 3 to 6 mice used in each experimental group (20 to 150 isolated DRG neurons from one mouse in each single experiment).

### 4.5. ROS Measurements

To measure cytoplasmic ROS levels, we used OxiSelectTM Intracellular ROS Assay Kit (Green Fluorescence; Cell Biolabs, San Diego, CA, USA). DRG neurons from C57BL/6J mice were cultured in either control or high glucose for 7 days. For high glucose solution, the concentration of glucose was increased to 25 mM. Following this, the neurons were incubated for one hour at 37 °C with medium containing DCFH-DA (1 mM) and subsequently washed three times with PBS. A Till Photonics single-cell fluorescence imaging system was used to take the florescent images of neurons at room temperature. For each image, we defined regions of interest and measured the mean fluorescent intensity. The background-subtracted fluorescence intensity values were compared in control and high glucose DRG neurons to determine the ROS level increases under high glucose condition in DRG neurons.

### 4.6. Immunocytochemistry

Isolated DRGs were fixed in 10% neutral formalin and embedded in paraffin blocks and sectioned at 5 microns. Deparaffinized sections of the ganglia were incubated with the primary goat TRPV1 antibody in dilution buffer (3% normal horse serum and 0.04 Triton-x; Santa Cruz, Cat. # sc-398417; dilution factor: 1:100) overnight at 4 °C followed by three washes in PBS. The sections were then incubated with the secondary antibody (dilution factor: 1:1000, Donkey-anti-goat biotin-conjugated antibody, Sigma-Aldrich, St. Louis, MO, USA). After the secondary antibody was removed by multiple PBS washes, the sections were treated with horseradish peroxidase-conjugated streptavidin. The color was developed using diaminobenzidine. DRG sections (6 sections from each mouse) were randomly selected from wild-type (*n* = 7) or *Ins2^+/Akita^* (*n* = 7) mice and TRPV1-expressing neurons were counted by blinded assayer using Image-Pro Premier 9.3 (Media Cybernetics, Rockville, MD, USA). 

### 4.7. Chemicals

All inorganic salts and buffers were purchased from Sigma (St. Louis, MO, USA). The TRPV1 antibody was from Santa Cruz (Cat. # sc-398417, Dallas, TX, USA). Fura-2AM (Cat. # F-1221) was from Thermo Fisher Scientific (Waltham, MA, USA).

### 4.8. Statistical Analysis

The SigmaPlot 12 software (Systat software, San Jose, CA, USA) was used to perform the statistical analyses. The unpaired *t* test was used to determine the significant difference between two groups of data with normally distributed populations and equal variances, whereas the Mann–Whitney Rank Sum Test was used to determine the significant difference between two groups of data with non-normally distributed populations and different variances. The significance level was set to <0.05. All data were presented as mean ± standard error of the mean (SEM).
